# Autonomic cardiac function in children and adolescents with long COVID: a case-controlled study

**DOI:** 10.1007/s00431-024-05503-9

**Published:** 2024-03-06

**Authors:** A. B. Delogu, C. Aliberti, L. Birritella, G. De Rosa, C. De Rose, R. Morello, N. Cambise, A. G. Marino, A. Belmusto, L. Tinti, A. Di Renzo, G. A. Lanza, D. Buonsenso

**Affiliations:** 1 Institute of Pediatrics, Pediatric Cardiology, Department of Woman and Child Health and Public Health, Fondazione Policlinico Universitario A. Gemelli IRCCS, Rome, Italy; 2 Università Cattolica del Sacro Cuore, Rome, Italy; 3 Institute of Pediatrics, Department of Woman and Child Health and Public Health, Fondazione Policlinico Universitario A. Gemelli IRCCS, Rome, Italy; 4 Department of Cardiovascular and Thoracic Sciences, Fondazione Policlinico Universitario A. Gemelli IRCCS, Rome, Italy; 5 Dipartimento Scienze della Salute della Donna, del Bambino e di Sanità Pubblica Università Cattolica del Sacro Cuore, Largo Agostino Gemelli 8, Rome, 00168 Italy

**Keywords:** Long COVID, Autonomic cardiac function, Heart rate variability, Cardiovascular, Children

## Abstract

Although the mechanisms underlying the pathophysiology of long COVID condition are still debated, there is growing evidence that autonomic dysfunction may play a role in the long-term complications or persisting symptoms observed in a significant proportion of patients after SARS-CoV-2 infection. However, studies focused on autonomic dysfunction have primarily been conducted in adults, while autonomic function has not yet been investigated in pediatric subjects. In this study, for the first time, we assessed whether pediatric patients with long COVID present abnormalities in autonomic cardiac function. Fifty-six long COVID pediatric patients (mean age 10.3 ± 3.8 y) and 27 age-, sex-, and body surface area-matched healthy controls (mean age 10.4 ± 4.5y) underwent a standard 12-lead electrocardiography (ECG) and 24-h ECG Holter monitoring. Autonomic cardiac function was assessed by time-domain and frequency-domain heart rate variability parameters. A comprehensive echocardiographic study was also obtained by two-dimensional echocardiography and tissue Doppler imaging. Data analysis showed that pediatric patients with long COVID had significant changes in HRV variables compared to healthy controls: significantly lower r-MSSD (root mean square of successive RR interval differences, 47.4 ± 16.9 versus 60.4 ± 29.1, *p* = 0.02), significant higher values VLF (very low frequency, 2077.8 ± 1023.3 versus 494.3 ± 1015.5 ms, *p* = 0.000), LF (low frequency, 1340.3 ± 635.6 versus 354.6 ± 816.8 ms, *p* = 0.000), and HF (high frequency, 895.7 ± 575.8 versus 278.9 ± 616.7 ms, *p* = 0.000). No significant differences were observed between the two groups both in systolic and diastolic parameters by echocardiography.

*  Conclusion*: These findings suggest that pediatric patients with long COVID have an imbalance of cardiac autonomic function toward a relative predominance of parasympathetic tone, as already reported in adult patients with long COVID. Further studies are needed to clarify the clinical significance of this autonomic dysfunction and demonstrate its role as a pathophysiological mechanism of long COVID, paving the way for effective therapeutic and preventive strategies.

**Table:** 

**What is Known:** *• Long Covid in children has been described globally, but studies have mostly focused on collecting the temporal evolution of persisting symptoms.*	
**What is New:** *• Cardiac autonomic imbalance toward a relative predominance of parasympathetic tone is a mechanism underlying Long Covid in children, as also described in adults.*

**What is Known:**

*• Long Covid in children has been described globally, but studies have mostly focused on collecting the temporal evolution of persisting symptoms.*

**What is New:**

*• Cardiac autonomic imbalance toward a relative predominance of parasympathetic tone is a mechanism underlying Long Covid in children, as also described in adults.*

## Introduction

Post-acute sequela of SARS-CoV-2 infection (PASC), also known as long COVID, is defined as the persistence of at least one initial symptom more than three months after initial infection, not explained by another etiology, with symptoms lasting more than 2 months [1, 2].

Long COVID condition, observed in a significant proportion of patients affected by COVID-19, consists of a broad range of manifestations affecting multiple organ systems, including unusual fatigue, post exertional malaise, cognitive dysfunction, and cardiorespiratory symptoms [3, 4]. This condition, initially described in adults, has now been documented also in children and adolescents [2, 5].

Despite a large number of studies and the description of various abnormalities, including organ injury, viral persistence, immune dysregulation, and autoimmunity, the mechanisms responsible for long COVID remain not completely understood [6, 7].

Autonomic dysfunction, however, is emerging as a potential relevant determinant or contributing factor to long COVID symptoms [8–13]. Previous studies, indeed, have shown, in particular, a significant impairment of cardiac autonomic function in adult patients with long COVID [14, 15]. However, there is no evidence of autonomic dysfunction in children with long COVID so far. This represents an important gap of current knowledge in pediatric long COVID, as cardiac autonomic dysfunction may be a potentially treatable condition.

In recent years, heart rate variability (HRV) has been recognized as a valuable and noninvasive tool to assess cardiac autonomic function in adults as well as in children [16, 17].

Therefore, in this study, we aimed to assess whether abnormalities in cardiac autonomic function are present in children and adolescents with long COVID.

## Materials and methods

### Study population

In this observational case–control study, we enrolled 56 consecutive pediatric patients (< 16 years of age) who were diagnosed having a long COVID at the post-COVID outpatient pediatric clinic of our hospital (Fondazione Policlinico Universitario A. Gemelli IRCCS, Rome, Italy) between April 2022 and April 2023. Patients were excluded in case of age > 16 years, history of congenital cardiac defects, or previous history of any other cardiac or respiratory tract disease.

Long COVID diagnosis was based on the most recent World Health Organization definition, for which the syndrome is a post COVID-19 condition in children and adolescents who experience symptoms occurring within 3 months from acute COVID-19 and lasting for at least 2 months [2]. The diagnosis was made after careful in-presence clinical assessment by a clinician expert in pediatric long COVID [17]. In children evaluated for persisting symptoms after SARS-CoV-2 infection in our post-COVID outpatient pediatric clinic, blood tests (including complete blood cell count, liver and kidney function, glucose, tests of thyroid function, celiac disease, antinuclear and extractable nuclear antigen antibodies (ANA and ENA), C-ANCA, and P-ANCA) were performed to exclude other diseases that might be the causes of persisting symptoms [17].

The inclusion criteria for the study were the presence of persistent or ex novo symptoms lasting at least 2 months which initially occurred within 3 months of acute COVID-19, according to the WHO definition above mentioned, and were present since no more than 9 months. We only included in this group children with microbiologically confirmed (based on a positive nasopharyngeal molecular test) SARS-CoV-2 infection. Patients were compared with a group of 27 healthy control subjects, matched to patients according to age, sex, and body surface area. Control children were a historical cohort of children referred in 2019 (before the pandemic) to our pediatric cardiology unit for a clinical check-up and found to have normal cardiac examination, electrocardiogram, and transthoracic Doppler echocardiography and underwent 24-h electrocardiogram with assessment of HRV.

All included subjects and their parents released their informed consent for treatment of clinical data, after being informed about the aim of the study, according to the principles of the Declaration of Helsinki.

Demographic characteristics, including age and sex, were recorded. All subjects underwent assessment of blood pressure (BP), heart rate (HR), body surface area (BSA), clinical history, and modifiable cardiovascular risk factors (hypertension, obesity, dyslipidemia, smoking, and sedentary habits). In all children with long COVID, a detailed medical history, concerning both the acute phase of SARS-CoV-2 infection and the post-COVID-19 persistent manifestations, was obtained. Primary outcome of this study was to assess HRV parameters in children with long COVID compared with a healthy control group. Secondary outcome was to compare echocardiographic findings in the same cohorts.

### Electrical and autonomic cardiac function

All participants underwent standard 12-lead electrocardiogram (ECG) and 24-h ECG Holter monitoring (HM), performed by 3-channel digital recorders (Medilog FD5-plus; Schiller, Milan, Italy).

The corrected QT interval (QTc) was calculated on standard ECG by the Bazett formula.

Cardiac autonomic function was assessed on 24-h Holter monitoring by heart rate variability (HRV). HRV parameters were obtained both in the time domain and frequency domain using the Medilog Darwin-2 software (Schiller Medilog, Milan, Italy) [18].

Time domain HRV parameters included: the standard deviation of normal-to-normal (NN) QRS intervals (SDNN), the mean of the standard deviations of all the NN intervals for each 5-min segments in the 24 h (SDNN-i), the standard deviation of the average NN intervals for each 5-min segments in the 24 h (SDANN), the root square of the mean of squares of successive NN interval differences (r-MSSD), and the percentage of successive NN intervals that differ by more than 50 ms (pNN50). Frequency domain HRV variables were assessed by fast Fourier transform spectral analysis, obtaining the amplitudes of the RR interval changes in the ranges of very low-frequency band (VLF, 0.0033–0.04 Hz), low-frequency band (LF, 0.04–0.15 Hz), and high-frequency band (HF, 0.15–0.40 Hz). VLF power is strongly influenced by parasympathetic nerve activity, as parasympathetic blockade considerably reduces it whereas it is not significantly influenced by sympathetic activity; other factors contribute to its genesis, including thermoregulatory mechanisms and renin–angiotensin and endothelial activity. LF power mainly reflects RR oscillation in the baroreceptor activity and is primarily influenced by parasympathetic nerve activity, although sympathetic function also has influence on its values, in particular in non-resting conditions. HF power, finally, is typically related to parasympathetic activity oscillation during respiratory cycles [19]. Accordingly, increased parasympathetic activity results in an increase of all frequency-domain HRV components. The average heart rate (HR) over the whole recording was also considered.

### Echocardiographic study

All subjects underwent a comprehensive echocardiographic study including two-dimensional echocardiography (2D Echo) and tissue Doppler imaging (TDI). All echocardiographic studies were performed using a Philips iE33 ultrasound system with a Philips X5-1 probe (Philips Medical System, Andover, Massachusetts, USA). Left ventricle (LV) dimension and function were assessed according to the pediatric recommendations) [20].

Standard echocardiographic parameters included LV end-diastolic (LVEDD) and end-systolic (LVESD) diameters, interventricular septum (IVS), and posterior wall (PW) diastolic thickness. Measurements were indexed for BSA. As surrogates of LV systolic function, we determined the LV shortening fraction (LVSF) on M-mode imaging, LV ejection fraction (LVEF) by biplane Simpson’s method, and the myocardial performance index (MPI). MPI is an index that incorporates both systolic (isovolumic contraction time (IVCT) and LV ejection time (LVET)) and diastolic (isovolumic relaxation time (IVRT)) time intervals. It is, therefore, an expression of both global systolic and diastolic LV function and is calculated with the following formula: IVCT + IVRT/LVET.

Left ventricular diastolic function was assessed by pulsed wave Doppler and TDI, obtaining the following parameters: 1) *E*/*A* wave ratio, i.e., the ratio between the peak of early diastolic Doppler mitral blood flow velocity (*E* wave) and the peak of late diastolic Doppler mitral blood flow velocity (*A* wave) and 2) septal and lateral *E*/*e*′ wave ratio, i.e., the ratio between the *E* wave peak and the peak of the early diastolic myocardial velocity (*e*′ wave) on tissue Doppler imaging.

Right ventricle (RV) systolic function was assessed by tricuspid annular plane systolic excursion (TAPSE), measured by two-dimensional-guided M-mode tracing, with the cursor optimally aligned along the direction of the tricuspid lateral annulus in the apical four-chamber view.

### Statistics

Data were analyzed with SPSS 23.0 statistical software (SPSS Chicago, IL, USA). Continuous variables are reported as means and standard deviations, while categorical variables as numbers and percentages. The normal distribution of variables was assessed by the Kolmogorov–Smirnov test. The comparisons of continuous variables were made with the analysis of variance (ANOVA), whereas categorical variables were compared by Fisher exact test. Statistical significance was set at a two-tailed *p* value < 0.05

## Results

### Study population

The main demographic and clinical characteristics of the 56 post-COVID patients and 27 healthy controls included in the study are reported in Table 1. Seven patients initially evaluated in our post-COVID clinic were excluded due to diagnosis of other conditions that might explain the persisting symptoms.

**Table 1:** Demographic and clinical characteristics of long COVID and healthy controls

	**Long COVID**	**Healthy controls**	***p***
Age (years)	10.3 ± 3.8	10.4 ± 4.5	0.87
Male sex	29 (51%)	19 (70%)	0.12
Weight (kg)	39.2 ± 16.5	43.2 ± 23.2	0.53
Height (cm)	141.2 ± 25.2	138.9 ± 30.4	0.77
BSA (m^2^^)^	1.2 ± 0.4	1.3 ± 0.4	0.24
SBP (mmHg)	104.2 ± 9.9	100.7 ± 10.5	0.42
DBP (mmHg)	59.4 ± 8.1	59.3 ± 10	0.98
MAP (mmHg)	74.4 ± 7.8	73.1 ± 6.8	0.58

*BSA* body surface area, *DBP* diastolic blood pressure, *MAP* mean arterial pressure, *SBP* systolic blood pressure

Data are expressed as mean ± standard deviation or number (%)

There were no significant differences in age, sex and main clinical features between the two groups. Mean age at enrollment was 10.3 ± 3.8 years in patients vs 10.4 ± 4.5 years in healthy subjects (*p* = 0.87); males were 51% of patients vs. 70% of healthy subjects (*p* = 0.12); BSA was 1.2 ± 0.4 m [2] for patients vs. 1.3 ± 0.4 m [2] for healthy subjects (*p* = 0.24).

During the acute phase of SARS-CoV-2 infection, the main symptoms of post-COVID-19 patients were fever (59%), headache (48%), rhinorrhea (39%), cough (21%), myalgia (21%), and fatigue (21%, defined as referred inability to perform the usual activities (including sport and school attendance) performed before SARS-CoV-2 infection), diarrhea (16%), hypo/anosmia (14%), dysgeusia (11%), joint pain (11%), dyspnea (9%), chest pain (7%), and cutaneous manifestations (7%). A diagnosis of multisystem inflammatory syndrome (MIS-C) associated with COVID-19 was made in one patient only (1/56; 2%) (Table 2A).

**Table 2:** Symptoms during the acute phase and follow-up in 56 patients with long COVID

A**. Symptoms during the acute phase**	
Fever	33 (59%)
Headache	27 (48%)
Rhinorrhea	22 (39%)
Cough	12 (21%)
Myalgias	12 (21%)
Fatigue	12 (21%)
Diarrhea	9 (16%)
Anosmia	8 (14%)
Dysgeusia	6 (11%)
Joint pain	6 (11%)
Dyspnea	5 (9%)
Chest pain	4 (7%)
Skin rashes	4 (7%)
MIS-C	1 (2%)
**B. Persistent COVID symptoms**	
Fatigue	26 (46%)
Persistent dyspnea	21 (38%)
Headache	13 (23%)
Myalgias	12 (21%)
Palpitations	10 (18%)
Chest pain	9 (16%)
Nausea	4 (7%)
Syncope	2 (4%)
Anosmia	1 (2%)
Dysgeusia	1 (2%)

The post-COVID-19 symptoms reported at enrollment were fatigue (46%), persistent dyspnea (38%), headache (23%), myalgia (21%), palpitations (18%), chest pain (16%), nausea/gastrointestinal distress (7%), syncope (4%), hypo/anosmia (2%), and dysgeusia (2%) (Table 2B). At clinical examination, lung and cardiac auscultation, abdominal clinical examination, and skin and hair inspection were normal in all children.

### Electrocardiogram and heart rate variability studies

The main results of standard ECG and Holter monitoring in the two groups are shown in Table 3.

**Table 3:** Comparison between long COVID and healthy controls for standard ECG, 24-h ECG Holter monitoring, and heart rate variability parameters

	**Long COVID**	**Healthy controls**	***p***
**Standard ECG**			
HR (bpm)	84.7 ± 21.4	87.00 ± 16.5	0.82
PR (ms)	135.9 ± 26.2	138.4 ± 6.2	0.84
QRS (ms)	86.1 ± 7.4	90.4 ± 5.2	0.22
QTc (ms)	393.4 ± 20.1	373.6 ± 13.8	0.04*
**24-h ECG Holter monitoring**			
Mean HR in 24 h (bpm)	87.4 ± 10.1	85.4 ± 16.4	0.59
PVCs	11(19%)	5(19%)	0.9
PACs	6(11%)	7(27%)	0.7
Time-domain HRV parameters*			
SDNN (ms)	136.3 ± 32.18	147.4 ± 43.8	0.24
SDNNi (ms)	68.6 ± 17.5	76.6 ± 27.7	0.21
SDANN (ms)	115.8 ± 30.8	123.6 ± 38.2	0.36
r-MSSD	47.4 ± 16.9	60.4 ± 29.1	0.02*
pNN50%	21.9 ± 12.3	26.5 ± 14.4	0.17
Frequency-domain HRV parameters*			
VLF (ms [2])	2078 ± 1023.3	494.3 ± 1015.5	0.000*
LF (ms^2^)	1340.3 ± 635.6	354.6 ± 816.8	0.000*
HF (ms^2^)	895.7 ± 575.8	278.9 ± 616.7	0.000*

*HR* heart rate, *HRV* heart rate variability, *PACs* premature atrial complexes, *PVCs* premature ventricular complexes, *QTc* corrected QT interval, *r-MSSD* root mean square of successive RR interval differences

^*^See text for definition of HRV parameters

On standard ECG, there were no significant differences between the two groups in HR, PR, and QRS intervals. QTc interval was slightly longer in long COVID patients compared to controls, but values were still within the normal range (393.4 ± 20.1 versus 373.6 ± 13.8 ms; *p* = 0.04).

On 24-h ECG Holter monitoring, no clinically relevant arrhythmias were recorded. Isolated premature atrial complexes (PACs) and premature ventricular complexes (PVCs) occurred in both groups, without significant differences in frequency, timing, and morphology.

HRV analysis showed no significant differences between long COVID patients and healthy controls for HRV parameters in the time domain except for r-MSSD, which showed lower values in long COVID patients (47.4 ± 16.9 vs. 60.4 ± 29.1 ms, *p* = 0.02).

On the other hand, most frequency-domain HRV parameters were significantly higher in long COVID patients compared to healthy controls (Table 3).

### Echocardiographic study

The results of the echocardiographic study are summarized in Table 4. There were no significant differences between long COVID and healthy controls in all key parameters of systolic and diastolic LV function.

**Table 4:** Main echocardiographic variables in long COVID and healthy controls

	**Long COVID**	**Healthy controls**	***p* value**
LVEED (mm)	42.1 ± 5.9	42.3 ± 6.8	0.91
LVESD (mm)	29 ± 7.1	26.3 ± 5.6	0.22
IVSd (mm)	6.9 ± 1.4	7.1 ± 1.5	0.6
PWd (mm)	5.5 ± 1.2	6.8 ± 1.7	0.002*
FS (mm)	39.1 ± 3.8	38.9 ± 6.8	0.19
EF Simpson (%)	64.2 ± 5.3	64.7 ± 4.5	0.76
E/A	1.7 ± 0.3	2 ± 0.7	0.1
E/E’	6.2 ± 1.3	5.9 ± 0.9	0.39
TAPSE (mm)	21.1 ± 3.6	22.9 ± 2.6	0.12
MPI = IVCT + IVRT/ET	0.5 ± 0.1	0.5 ± 0.03	0.8

*E/A* ratio of early diastolic mitral inflow velocity (*E*) to late diastolic mitral inflow velocity (*A*), *E/E*′ ratio of early mitral Doppler peak flow (*E*) to early diastolic myocardial velocity (*E*′), *EF* ejection fraction, *ET* ejection time, *FS* fractional shortening, *IVCT* isovolumic contraction time, *IVRT* isovolumic relaxation time, *IVSd* interventricular septum end-diastolic diameter, *LVEDD* left ventricular end-diastolic diameter, *LVESD* end-systolic diameter, *MPI* myocardial performance index, *PWd* posterior wall end-diastolic diameter, *TAPSE* tricuspid annular plane systolic excursion

Data are expressed as mean ± standard deviation

## Discussion

To the best of our knowledge, in this study, we report, for the first time, results on cardiac autonomic function in a pediatric cohort with long COVID in comparison to healthy controls, using HRV analysis, an established proxy measure of cardiac autonomic nervous system activity [16]. We found that children and adolescents with long COVID have altered values of HRV compared to healthy subjects. We therefore believe that this study can shed light on the mechanisms responsible for this condition, which could involve autonomic dysfunction as previously described in adults.

In this study, we also found a slightly longer QTc interval in long COVID patients. However, the values were largely within the normal range and seemed, therefore, of scarce clinical relevance. Whether this mildly longer QTc interval was in some way related to some cardiac involvement in the acute COVID illness or was just a casual result in our study deserves investigation in larger studies.

In our pediatric patients with long COVID, cardiac autonomic sympatho-vagal imbalance was, on the whole, characterized by a prevalence of parasympathetic activity, as indicated by higher power levels, compared to healthy controls, of all frequency-domain variables.

In contrast with the frequency-domain data, however, the time-domain variable RMSSD was unexpectedly found lower in patients compared to healthy controls. The reason for this apparent discrepancy is not clear, but the lower RMSSD might reflect an abnormality of the non-autonomic regulation of the sinus cycle resulting in a reduction of immediate variability of RR interval, indicated by the lower RMSSD values, in a context of general increase of HRV. Alternatively, the increase of most HRV components might mainly result from an increased activity of parasympathetic function related to the respiratory cycle, by which, however, RMSSD is poorly influenced. Finally, we cannot exclude that the different behavior of RMSSD is merely related to chance. As this is the first study addressing these parameters in children, we do not have comparisons for our findings, and this did not even allow us to derive a sample size calculation. Future studies should further assess this aspect.

Independently of the actual reasons for this apparent discordance, the anomalous increase in parasympathetic activity of cardiac autonomic function detected in long COVID patients might be involved in their symptoms, in particular those related to cardiovascular function, including palpitations and dyspnea.

These findings have relevant practical implications, considering that, for a long time, long COVID was not considered a particularly relevant disease in children [5]. Even in the WHO definition of pediatric long COVID [2] anxiety is reported as one of the most common symptoms, raising doubts and debates among family associations and experts about whether mental health disorders are the cause of this condition or a consequence of a disrupted daily life due to debilitating symptoms [21]. Our findings further reinforce previous preliminary reports that, even in children, persisting post-acute symptoms are related to measurable biologic variables rather than psychological conditions only [22–27]. The results of our study, indeed, suggest that an anomalous increase in parasympathetic activity of the cardiac autonomic nervous system may constitute a pathophysiologic measurable mechanism for long COVID in children, opening opportunities for some potential causal forms of management (for example, a recent virtual meeting of the International Post-COVID Condition in Children Collaboration [28, 29] discussed the possibility of design trials to evaluate the role of ivabradine in children with long covid with evidence of dysautonomia [30]).

Recent studies in adults have brought new data about the potential link between autonomic dysfunction and some long COVID symptoms, particularly those related to the cardiovascular and respiratory systems [8–13, 31]. Postural orthostatic tachycardia syndrome (POTS) is the most frequent form of autonomic dysfunction reported in adults with long COVID, but also orthostatic hypotension and neurocardiogenic syncope have been reported [32, 33]. Likewise, fatigue and airway disorders in long COVID patients are other symptoms that have been associated with dysautonomia [12]. In particular, the latest studies suggest that SARS-CoV-2 may induce vagus nerve inflammation followed by autonomic dysfunction, which might contribute to critical disease courses and dysautonomia observed in long COVID [34].

Our data show that, on the whole, children with long COVID present a parasympathetic overtone, which might contribute to the symptoms of patients by several ways [35], including changes in the blood flow resulting in abnormal muscle, lung, and brain perfusion [22–26], leading to some of the most common and impactful long COVID symptoms, like fatigue, muscle pain, exercise intolerance, and dyspnea as well as cognitive symptoms like brain fog.

This study represents the first evidence of cardiac autonomic dysfunction with increased parasympathetic tone in children with long COVID and, if confirmed in other independent cohort, might have important clinical implications for therapeutic and preventive strategies. In addition, further research is needed to understand the clinical significance of the increased parasympathetic tone, investigating the mechanisms that lead to neuropathy and its consequences, and whether this damage is reversible (a follow-up assessment of these patients is currently ongoing). Of note, in a recent report, an adolescent with long COVID and evidence of lung hypoperfusion, abnormal cardiopulmonary exercise testing (CPET), and autonomic dysfunction based on HRV data had full recovery after treatment with anticoagulants which likely improved a thrombotic-related lung hypoperfusion, and ivabradine, which likely contributed to improved HRV through modulation of sinus node response to autonomic nervous system activity [25]. Our data add further information to the changes in cardiac autonomic function that patients with a long COVID syndrome may develop, suggesting that standardized assessment of autonomic function may be helpful in these children.

This study has limitations. We enrolled a relatively small number of patients. Furthermore, this being a single-center study, its generalizability might be limited. In addition, although our patients underwent a large variety of specific tests including lung SPECT and CPET [36], due to resource constraints we failed to collect all tests for all patients, and therefore, we could not assess the association of cardiac autonomic dysfunction with them. Last, to be sure that the controls had not contracted a subclinical SARS-CoV-2 infection, control groups were not selected from the same time period as individuals with SARS-CoV-2 infection, which may also have introduced some bias.

## Conclusion

In conclusion, our data suggest that also children with long COVID have changes in cardiac autonomic activity, which are characterized by a significant increase of parasympathetic autonomic function. Therefore, it is reasonable to speculate about a possible role of autonomic dysfunction as potential mechanism underlying long COVID in children as well as in adults. These findings may form the basis for investigation of the pathophysiologic mechanism(s) of autonomic dysfunction and possible effective therapeutic and preventive strategies in long COVID-19 children.

## Data Availability

Dataset is available upon request to the corresponding author.

## Abbreviations

PASC: Post-acute sequela of SARS-CoV-2 infection
HRV: Heart rate variability
BP: Blood pressure
HR: Heart rate
BSA: Body surface area
TDI: Tissue Doppler imaging
LV: Left ventricle
LVEDD: Left ventricle end-diastolic diameters
LVESD: Left ventricle end-systolic diameters
IVS: Interventricular septum
PW: Posterior wall
LVSF: Left ventricle shortening fraction
LVEF: Left ventricle ejection fraction
IVCT: Isovolumic contraction time
LVET: Left ventricle ejection time
IVRT: Isovolumic relaxation time

## Declarations

### Disclaimer

The funder had no role in the development of the project nor in the study design, data collection, data analysis, interpretation of results or writing of the report. DB had full access to dataset and had final decision to submit for publication. All authors had full access to all the data in the study and accept responsibility for the decision to submit for publication.
